# A green method to leach vanadium and chromium from residue using NaOH-H_2_O_2_

**DOI:** 10.1038/s41598-017-18918-2

**Published:** 2018-01-11

**Authors:** Hao Peng, Zuohua Liu, Changyuan Tao

**Affiliations:** 1 College of Chemistry and Chemical Engineering, Yangtze Normal University, Chongqing, 400044 China; 2 College of Chemistry and Chemical Engineering, Chongqing University, Chongqing, 400044 China

## Abstract

Hydrogen peroxide as an oxidant was applied in leaching of vanadium and chromium in concentrated NaOH solution. Under the optimal reaction conditions (the liquid to solid ratio of 4.0 ml/g, residue particle size of <200 mesh, the mass ratio of NaOH-to-residue of 1.0 g/g, the volume ratio of H_2_O_2_-to-residue of 1.2 ml/g, reaction temperature of 90 °C and reaction time of 120 min), the leaching efficiency of vanadium and chromium could reach up to 98.60% and 86.49%, respectively. Compared with the current liquid-phase oxidation technologies, the reaction temperature was 90–310 °C lower, and the NaOH concentration of the reaction medium is lower by more than 50 wt% (the mass ratio of NaOH-to-residue of 1.0 g/g equals to concentration of 20 wt%). The kinetics study revealed that leaching process of chromium and vanadium were interpreted with shrinking core model under chemical reaction control. The apparent activation energy of chromium and vanadium dissolution was 22.19 kJ/mol and 6.95 kJ/mol, respectively.

## Introduction

Vanadium is an important strategic metal in the manufacturing of iron and steel, non-ferrous metals and petrochemical industry, due to its excellent mechanical, electrochemical, catalytic, magnetic and other physicochemical properties^1–3^. Many hydrometallurgical processes have been proposed to recover vanadium and chromium^4–9^, but the problems associated with the high temperature salt roasting technologies, including the low overall resource utilization efficiency, the high energy consumption, and the environmental pollution, are still remain unsolved.

The liquid-phase oxidation (LPO) technologies had been developed to improve the resource utilization efficiency and meet more stringent environmental regulations. The sub-molten salt (SMS) technology, one of LPO technologies, had been successfully applied to leaching vanadium and chromium in concentrated alkali solution^10–13^. In this new LPO method, the leaching efficiency of vanadium and chromium could reach up to 95% and 90%, respectively. In addition, no hazardous gas or toxic tailings were discharged during the process compared with salt-roasting technology. The process needed high alkaline consumption, high energy cost and also the equipment corrosion was an big challenge due to the high causticity of the reaction medium (usually above 70 wt%).

Some other ways were conducted to improve the leaching efficiency of vanadium and chromium. Oxidizing substances like MnO_2_ and KClO_3_ were added to enhancing the leaching of vanadium in low valence during the leaching process^14,15^. Electricity as an environmental-friendly resource was also introduced to intensify the leaching process^5,16,17^. This paper presented a method that utilized H_2_O_2_ as an oxidant to oxidize vanadium and chromium in low valent in concentrated NaOH solution during the leaching process. The technology principles were discussed and the effects of the mass ratio of NaOH-to-residue, the volume ratio of H_2_O_2_-to-residue, reaction temperature and reaction time on the leaching efficiency of vanadium and chromium were systematically investigated.

## Results and Discussion

### Technology principle

In a vanadium and chromium residue particle, V, Cr, and Fe primarily existed in the central area as spinel (Fe(V,Cr)_2_O_4_), while Si primarily existed in the surrounding area as silicate (SiO_2_).

In concentrated NaOH solution, silicate could readily react with NaOH, exposing the spinel to the reaction medium. In the presence of H_2_O_2_, the spinel can be oxidized to produce water-soluble vanadate and chromate, as described below:

1 $$2{\rm{FeO}}\cdot {{\rm{V}}}_{2}{{\rm{O}}}_{3}+12{\rm{NaOH}}+5{{\rm{H}}}_{2}{{\rm{O}}}_{2}\to {{\rm{Fe}}}_{2}{{\rm{O}}}_{3}+11{{\rm{H}}}_{2}{\rm{O}}+4{{\rm{Na}}}_{3}{{\rm{VO}}}_{4}$$

2 $$2{\rm{FeO}}\cdot {{\rm{Cr}}}_{2}{{\rm{O}}}_{3}+8{\rm{NaOH}}+7{{\rm{H}}}_{2}{{\rm{O}}}_{2}\to {{\rm{Fe}}}_{2}{{\rm{O}}}_{3}+11{{\rm{H}}}_{2}{\rm{O}}+4{{\rm{Na}}}_{2}{{\rm{CrO}}}_{4}$$

**Figure 1:**
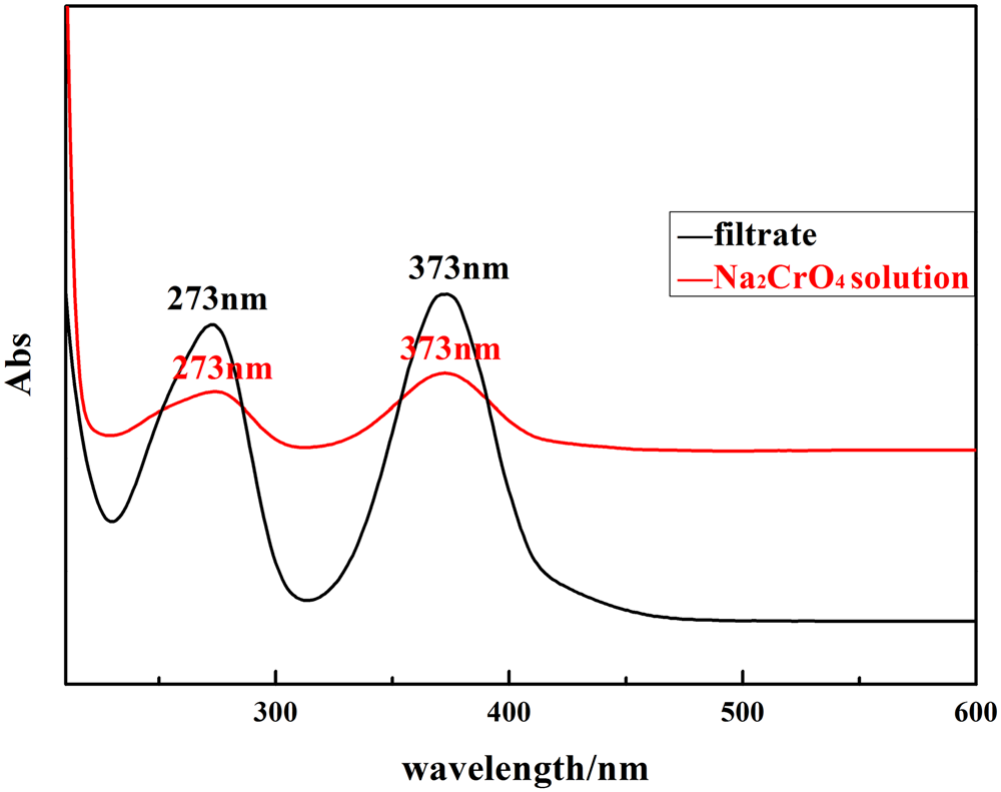
UV adsorption curve of filtrate and Na_2_CrO_4_ solution.

The UV adsorption of the filtrate and standard Na_2_CrO_4_ solution were measured using a UV–Vis spectrophotometer (TU-1901, Beijing Persee, China). The results were shown in Fig. 1. The filtrate was found to exhibit adsorption curve at 273 nm and 373 nm, which was the same with the standard Na_2_CrO_4_ solution. The results indicated that the chromium in low valent, Cr^3+^, was oxidized to chromium in high valent, CrO_4_^2−^, during the leaching process. So was vanadium, as vanadium was easy to be oxidized than chromium.

### Oxidative leaching of vanadium and chromium

#### Effect of mass ratio of NaOH-to-residue

**Figure 2:**
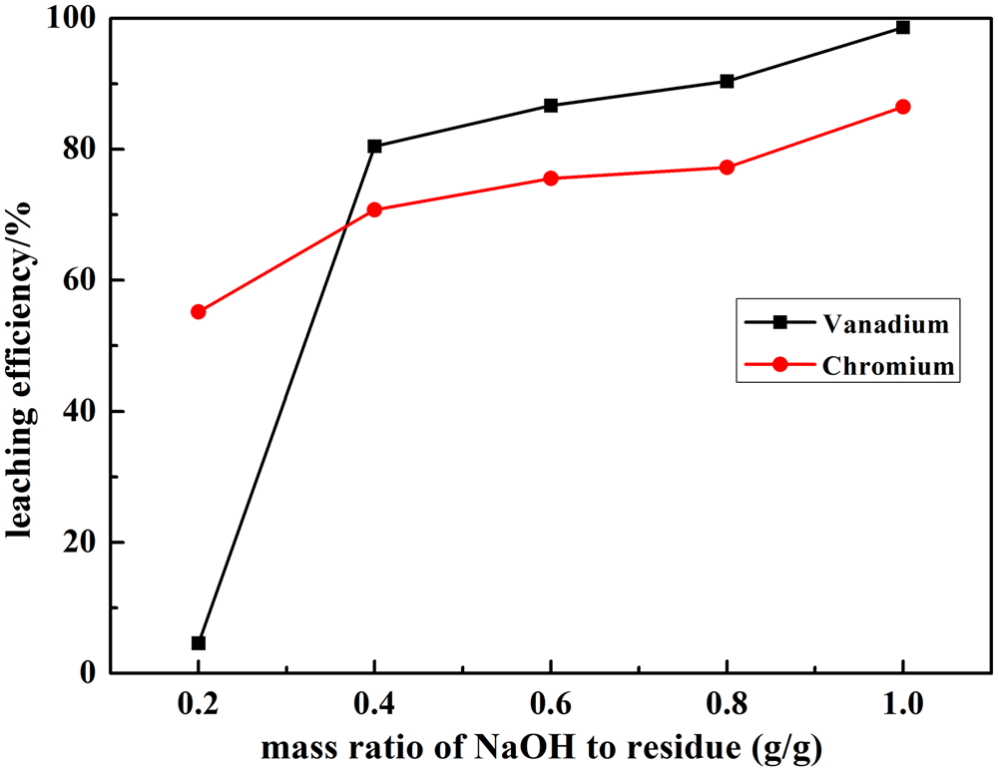
Effect of the mass ratio of NaOH-to-residue on leaching efficiency of vanadium and chromium.

The effect of mass ratio of NaOH-to-residue on the leaching efficiency was preferentially examined under the following conditions: the liquid to solid ratio of 4.0 ml/g, residue particle size of < 200 mesh, the volume ratio of H_2_O_2_-to-residue of 1.2 ml/g, reaction temperature of 90 °C, reaction time of 120 min. The leaching efficiency of vanadium and chromium over mass ratio of NaOH-to-residue were summarized in Fig. 2.

The results shown in Fig. 2 described that the leaching efficiency of vanadium and chromium both exhibited significant increase with the increase of the mass ratio of NaOH-to-residue (MR) from 0.2 g/g to 1.0 g/g. The leaching efficiency of vanadium and chromium was much higher with the addition of H_2_O_2_^5^. The reaction activity of the hydroxide ions dramatically increased with the increase of MR and enabled the oxidation reactions to be much more thermodynamics favorable^18^. When the MR was less than 0.4 g/g, the leaching efficiency increased sharply, and after that increased slightly. With the increase of MR, the medium viscosity increased, and the salting-out effect would be greatly intensified, resulting in significant decrease of the mass transfer efficiency^19^. It was indicated that high MR was more beneficial for the oxidation reactions in terms of thermodynamic consideration, while unfavorable for the reaction kinetics. According to Fig. 2, the increase in the activity had more significant impact on the leaching of vanadium and chromium in comparison with the decrease of oxidant solubility and mass transfer efficiency during the leaching process. Thus, the mass ratio of NaOH-to-residue of 1.0 g/g was chosen for the following experiments.

#### Effect of volume ratio of H_2_O_2_-to-residue

**Figure 3:**
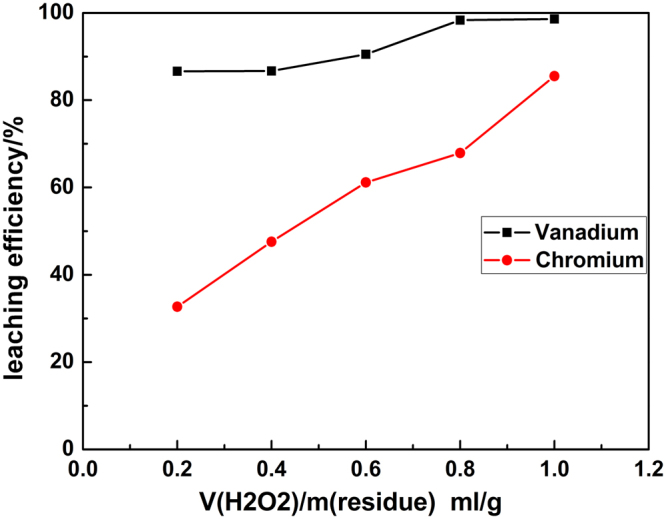
Effect of the volume ratio of H_2_O_2_-to-residue on leaching efficiency of vanadium and chromium.

The effect of volume ratio of H_2_O_2_-to-residue on the leaching efficiency was preferentially investigated under the following conditions: the liquid to solid ratio of 4.0 ml/g, residue particle size of < 200 mesh, the mass ratio of NaOH-to-residue of 1.0 g/g, reaction temperature of 90 °C, reaction time of 120 min. The leaching efficiency of vanadium and chromium over volume ratio of H_2_O_2_-to-residue were summarized in Fig. 3.

Figure 3 showed that the volume ratio of H_2_O_2_-to-residue (VR) had a small influence on leaching efficiency of vanadium, which just increased from 86.62% to 98.60% with the increase in the VR from 0.2 ml/g to 1.0 ml/g. The result was part of agreement with Yang *et al*.^14^.

The leaching efficiency of chromium exhibited a significant increase with the increase of VR from 0.2 ml/g to 1.0 ml/g. During the leaching process, H_2_O_2_ was mainly used to oxidize chromium in low valent to water-soluble chromate (CrO_4_^2−^). In other words, leaching of chromium was much more depend on the addition of H_2_O_2_ than vanadium.

#### Effect of reaction temperature

**Figure 4:**
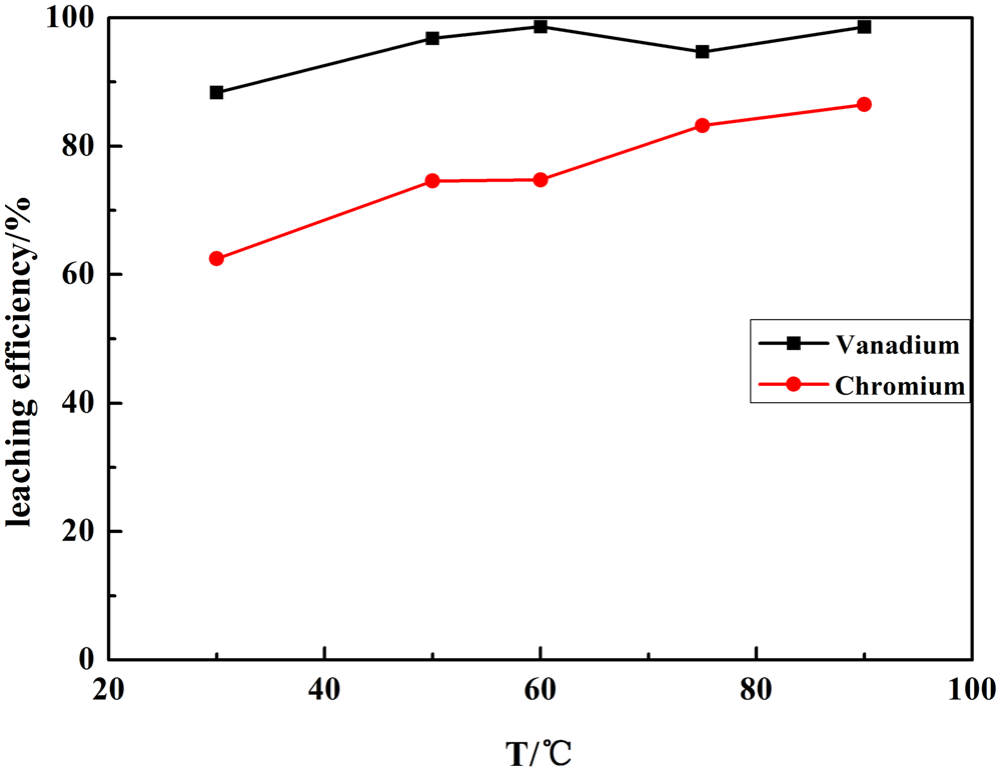
Effect of temperature on leaching efficiency of vanadium and chromium.

The reaction temperature was another important parameter that had significant influence on the leaching of chromium from both thermodynamic and kinetic consideration. Figure 4 summarized the effect of temperature on leaching efficiency of vanadium and chromium under the standard conditions: the liquid to solid ratio of 4.0 ml/g, residue particle size of < 200 mesh, the mass ratio of NaOH-to-residue of 1.0 g/g, the volume ratio of H_2_O_2_-to-residue of 1.0 ml/g, reaction time of 120 min.

The results indicated that the effect of temperature on the leaching efficiency of vanadium exhibited an analogously parabolic characteristic. This behavior occurred because as the temperature increased from 30 °C to 60 °C, the medium viscosity dramatically decreased. It was indicated that a higher temperature favors the mass transfer, considering that medium viscosity had a decisive impact on mass transfer once other conditions are specified

The leaching efficiency of chromium was increased from 62.45% to 86.49% with the increasing of temperature from 30 °C to 90 °C. As an important oxidant, mass transfer efficiency of H_2_O_2_ was vital for the control of the leaching kinetics, and also the leaching efficiency of vanadium was little gap in 60 °C and 90 °C. Thus, a temperature of 90 °C was chosen for the following experiments.

#### Effect of reaction time

**Figure 5:**
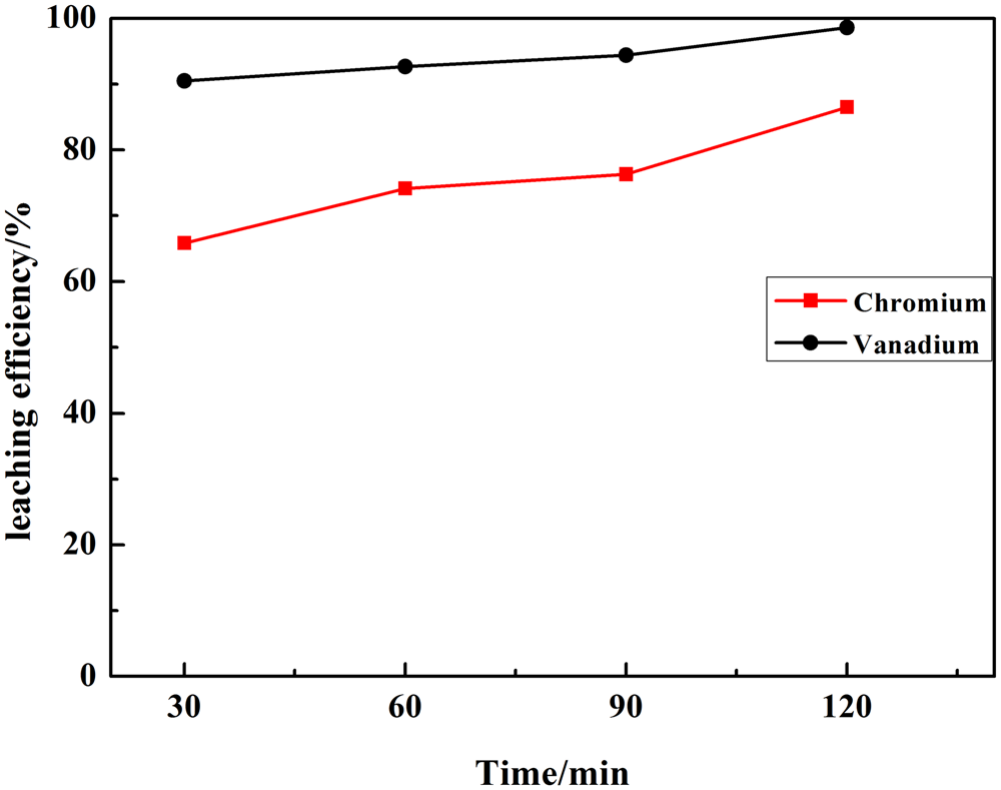
Effect of reaction time on leaching efficiency of vanadium and chromium.

The effect of reaction time on the leaching efficiency was preferentially studied under the following conditions: the liquid to solid ratio of 4.0 ml/g, residue particle size of < 200 mesh, the mass ratio of NaOH-to-residue of 1.0 g/g, the volume ratio of H_2_O_2_-to-residue of 1.2 ml/g, reaction temperature of 90 °C. The leaching efficiency of vanadium and chromium over reaction time were summarized in Fig. 5.

Figure 5 showed that the leaching efficiency of vanadium and chromium increased significantly. The leaching efficiency of vanadium and chromium was 98.60% and 86.49%, respectively, after reaction time of 120 min. Further reaction time might increase the leaching efficiency. In terms of energy efficiency, equipment corrosion alleviation, reaction time of 120 min was selected as the optimal condition as the leaching efficiency of vanadium and chromium was much high.

### Kinetics analysis

During the hydrometallurgy process, the leaching process mostly follows the shrinking core model. Three equations were used to describing the kinetic models^5,20^.

(1) Liquid boundary layer diffusion

3 $$X=kt$$

(2) Solid product layer diffusion control

4 $$1-2X/3-{(1-X)}^{2/3}=kt$$

(3) Chemical reaction control

5 $$1-{(1-X)}^{1/3}=kt$$

6 $$k=6{{\rm{\mu }}\mathrm{MDc}}_{{\rm{A}}}/({{\rm{\rho }}r}^{2})$$

where *X* is the leaching efficiency of chromium, *k* is the overall rate constant, min^−1^, *μ* is the stoichiometric coefficient, *M* is the molecular mass of the solid reactant, g/mol, *D* is the diffusion coefficient, m^2^/min, *c*_*A*_ is the NaOH concentration, mol/L, ρ is the density of the particle, g/mL, *r* is the radius of residue particle, m.

**Figure 6:**
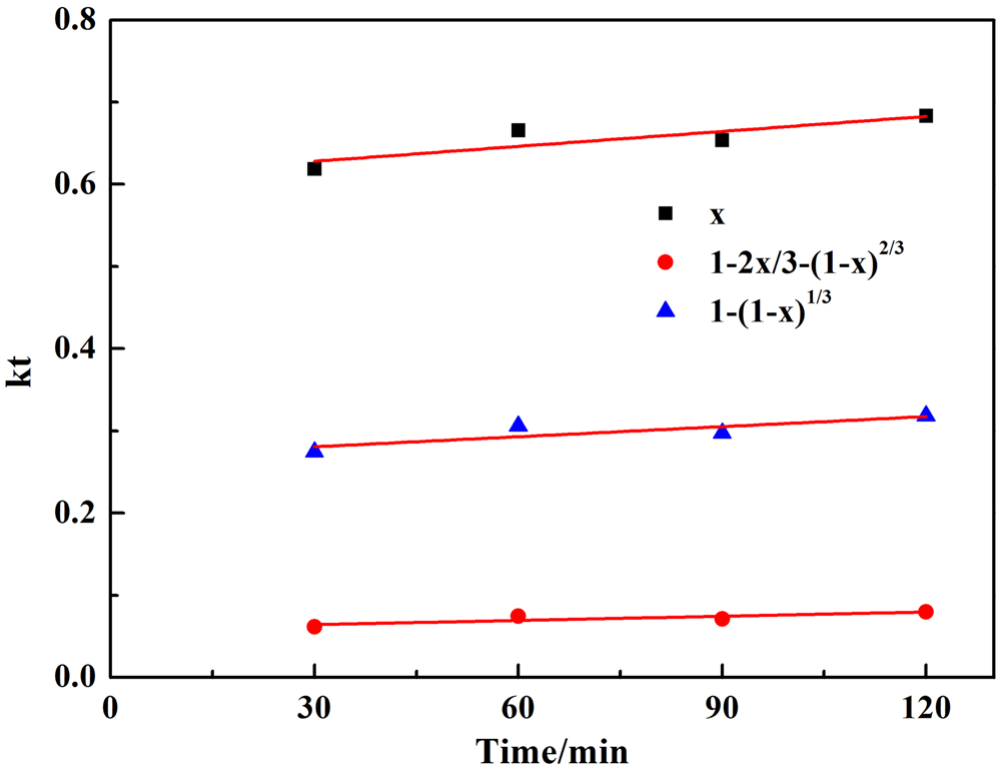
Chromium leaching rate vs time fitted by three kinetics equations at 90 °C.

The experimental data was fitted into Eqs (3–5) to determine the kinetic parameters and rate-controlling step in the process showed in Fig. 6. The results showed Eq. (5) fitted the experimental data perfectly. Therefore, the chemical reaction was the controlling step for the leaching process. So Eq. (5) was used to express the shrinking core model.

**Figure 7:**
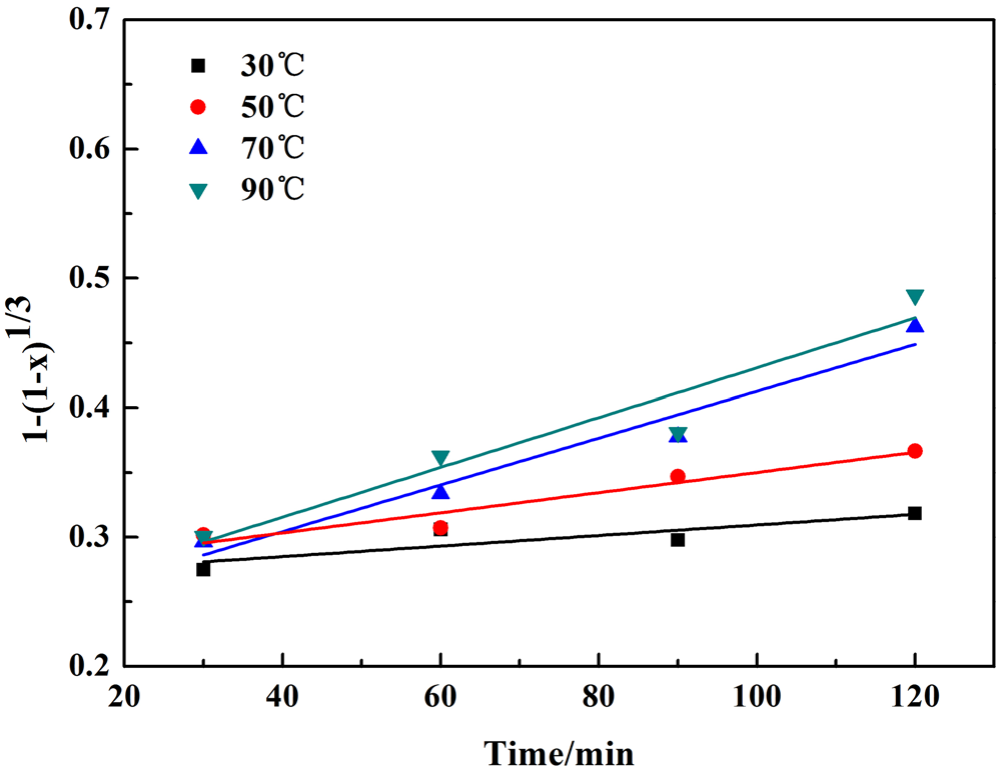
Plot of leaching kinetics of chromium at various reaction temperatures.

**Figure 8:**
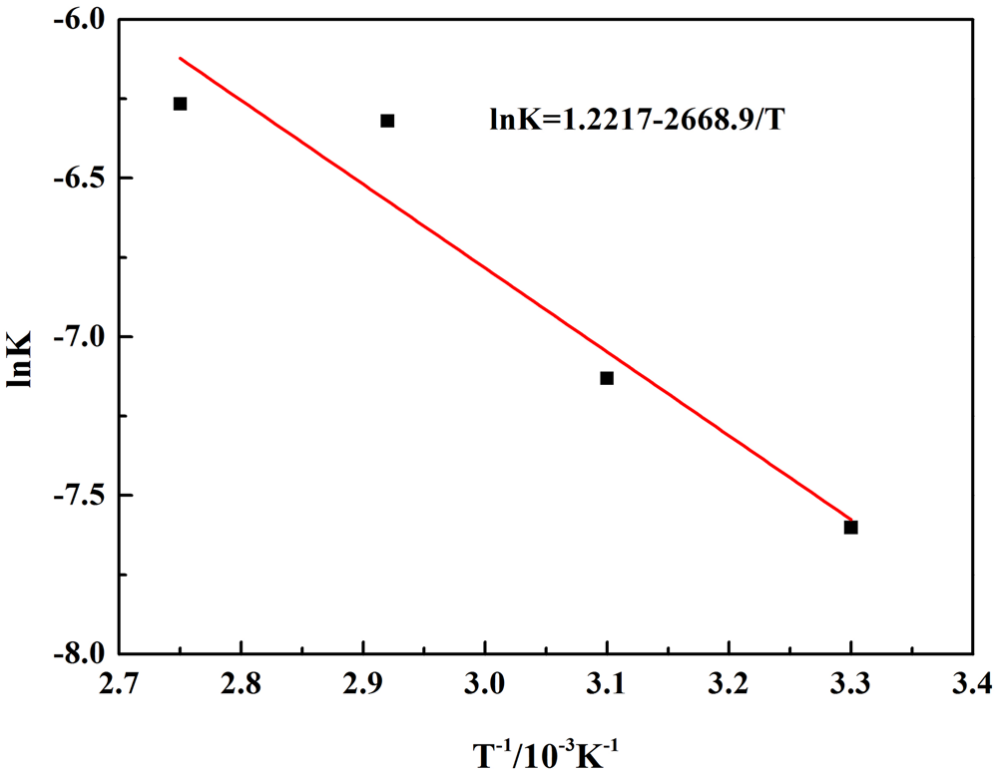
Natural logarithm of reaction rate constant versus reciprocal temperature of chromium.

The reaction rates of chromium at different temperatures were fitted in Fig. 7, and the value of K was obtained, where K was the reaction rate constant corresponding to the slopes of the straight lines. Then the specific apparent activation energy could be calculated based on the Arrhenius equations, the result showed in Fig. 8.

7 $${\rm{lnK}}={\rm{lnA}}-{\rm{Ea}}/({\rm{RT}})$$

where Ea is the apparent activation energy, A is the pre-exponential factor, and R is the molar gas constant.

The apparent activation energy of chromium leaching out was calculated to be 22.19 kJ/mol, which was much smaller than 50.27 kJ/mol calculated by Liu *et al*.^10^.

**Figure 9:**
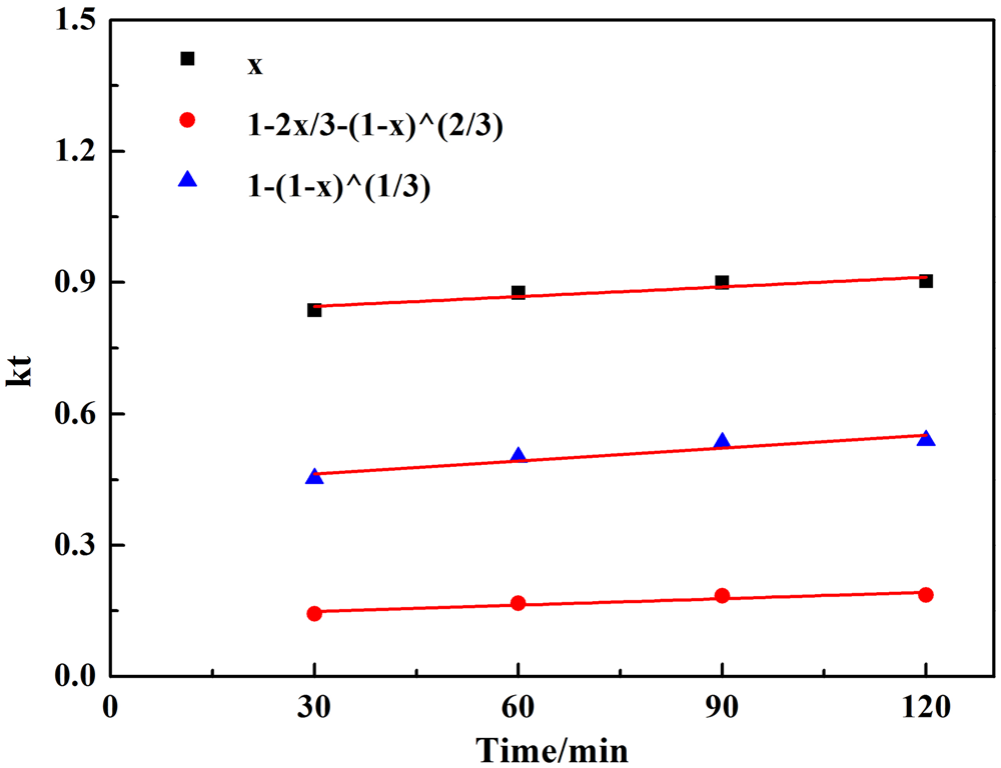
Vanadium leaching rate vs time fitted by three kinetics equations at 90 °C.

Similarly, to reveal the controlling step of the vanadium leaching, the experiment data was also fitted into Eqs (3–5), as shown in Fig. 9. The results showed that Equation (5) gave very good linear relationship. It was meant that the chemical reaction was the controlling step for the leaching process of vanadium.

**Figure 10:**
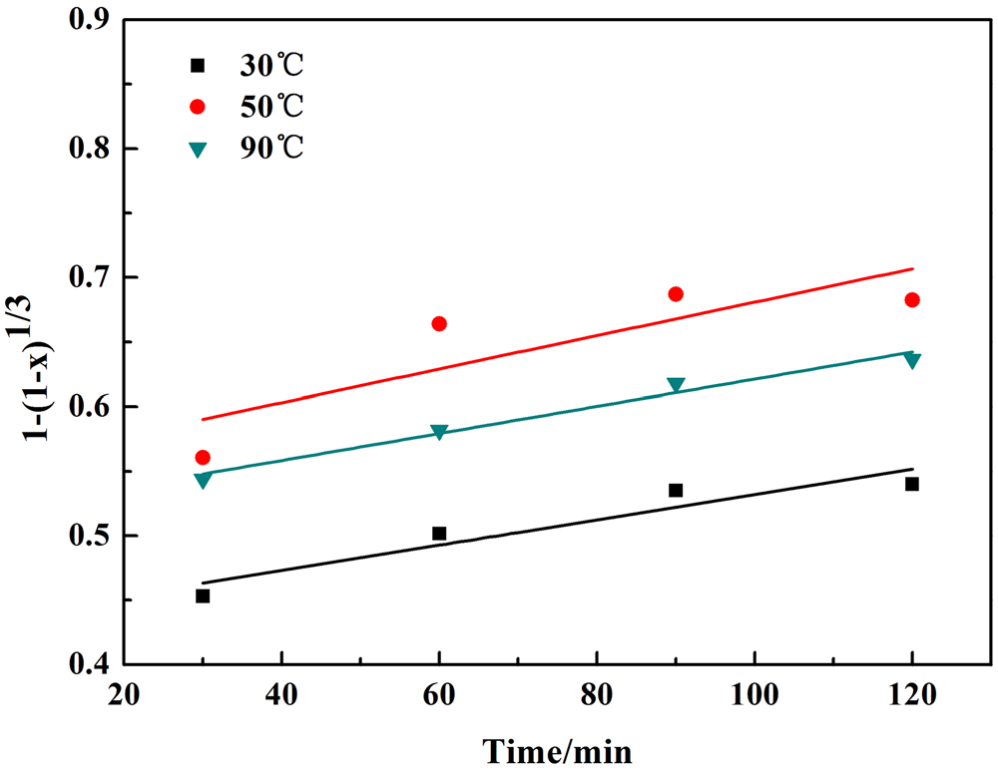
Plot of leaching kinetics of vanadium at various reaction temperatures.

**Figure 11:**
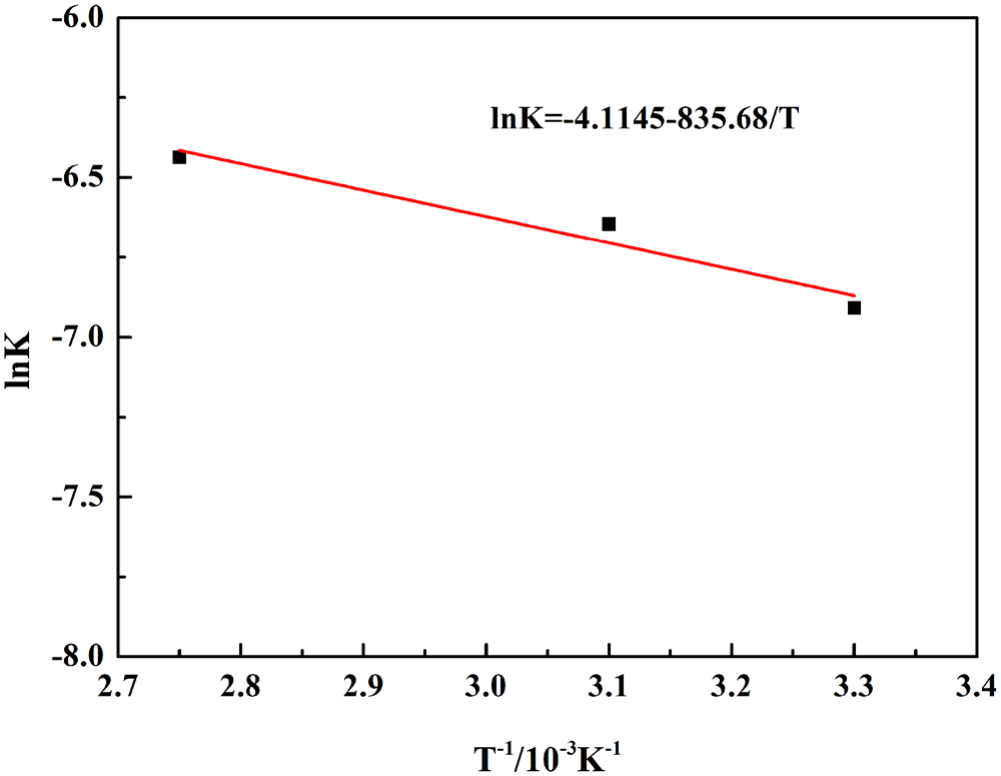
Natural logarithm of reaction rate constant versus reciprocal temperature of vanadium.

The leaching efficiency of vanadium at different temperatures was fitted in Fig. 10 and the apparent activation energy of vanadium leaching was calculated to be 6.95 kJ/mol (Fig. 11). The value was lower that of 10.08 kJ/mol^5^.

### Residue morphology before and after leaching

XRD (X-ray diffraction) analysis of the raw residue and leaching residue were conducted to analyze the change. The results had been discussed in our previous works^4^. The results showed that after leaching, the main content phases disappeared and only peak of SiO_2_ left. It was meant that vanadium and chromium in the residue were leached out, which was consistent with the results discussed above.

## Conclusions

1. Hydrogen peroxide as an oxidant was applied in leaching of vanadium and chromium in concentrated NaOH solution It was an environmental-friendly technology which was benefit than others (seen in Table 1). Under the optimal reaction conditions, the leaching efficiency of vanadium and chromium could reach up to 98.60% and 86.49%, respectively. Compared with the current liquid-phase oxidation technologies, the reaction temperature was 90–310 °C lower, and the NaOH concentration of the reaction medium is lower by more than 50 wt% (the mass ratio of NaOH-to-residue of 1.0 g/g equals to concentration of 20 wt%), showing substantial advantages in terms of energy efficiency, equipment corrosion alleviation and prospects for industrial application.

**Table 1:** Difference between NaOH-H2O2 method and others.

*technology*	*advantages*	disadvantages
sulfuric acid leaching	high leaching efficiency	high acid consumptionand high impurities
salt-roasting	easily leach out and high leaching efficiency	Toxic gases, non-environmental-friendly
sub-molten salt (SMS) technology	high leaching efficiency	high alkaline concentration, high process temperature and high ionic strength
Electro-oxidation technology	environmentally-friendly, and offered good selectivity for vanadium	low leaching efficiency for chromium
this work	High leaching efficiency of both vanadium and chromium; environmental-friendly
2. To obtain the optimal reaction conditions, the effects of various reaction parameters were systematically investigated. The leaching efficiency of vanadium was found to exhibited an analogously parabolic characteristic with the increase in reaction temperature; in addition, the increase in the mass ratio of NaOH-to-residue, the volume ratio of H_2_O_2_-to-residue, reaction time favored the leaching process, with the optimal reaction condition determined as the liquid to solid ratio of 4.0 ml/g, residue particle size of <200 mesh, the mass ratio of NaOH-to-residue of 1.0 g/g, the volume ratio of H_2_O_2_-to-residue of 1.2 ml/g, reaction temperature of 90 °C and reaction time of 120 min.
3. Leaching kinetic of chromium and vanadium were both controlled by chemical reaction. The apparent activation energy of chromium and vanadium dissolution was 22.19 kJ/mol and 6.95 kJ/mol, respectively.

## Methods

### Materials

The vanadium and chromium residue, supplied by Pangang Group Co., Ltd.,Chengdu, China, was precipitated from a waste water contained vanadium and chromium in low valence in an iron and steel mill. Before the experiment, the residue was first dried in oven overnight, followed by dry-sieving to obtain particles particle size of <200 mesh. The chemical composition of the residue is listed in Table 2.

**Table 2:** Composition of vanadium and chromium residue (%, wt).

Component	O	Cr	Si	Na	S	V
Amount (wt.%)	41.09	14.36	12.02	9.76	12.02	1.63
**Component**	**Ca**	**Cl**	**Fe**	**K**	**Mg**	
Amount (wt.%)	1.42	4.09	0.33	0.29	0.20	

All the reagents including sodium hydroxide, hydrogen peroxide used for leaching and sulfuric acid, phosphoric acid, ammonium ferrous sulfate, hexamethylenetetramine, potassium permanganate, and N-phenylantharalinic used in chemical analysis were analytical grade. Deionized water used in the experiments was produced by water purification system (HMC-WS10).

### Apparatus and procedures

All experiments were performed in a glass beaker with a thermostatic mixing water bath pot.

A predetermined amount of NaOH and deionized water was added to the beaker to produce homogeneous slurry under constant stirring. The slurry was heated to a predetermined temperature. Next, the residue was added to the reactor, and the addition of H_2_O_2_ was manually slow. After the required reaction time, the leachate was separated from the residue by vacuum filtration.

Titration with ammonium ferrous sulfate was used to determine the concentration of vanadium and chromium in the filtrate^21^.
